# Supplementary material for: Activation of Cannabinoid Type 2 Receptor in Microglia Reduces Neuroinflammation through Inhibiting Aerobic Glycolysis to Relieve Hypertension
Source: Biomolecules. 2024 Mar 11;14(3):333. doi: 10.3390/biom14030333 (PMC10967819; doi:10.3390/biom14030333)
Supplement: Supplementary file 1 [file biomolecules-14-00333-s001.zip › biomolecules-2878643-Supplementary Figure S1-S9 and Table S1.pdf]

Supplimentary materials

The mice were continuously administered Ang Ⅱ for 28 days, and the cerebral cortex tissues were collected at the end of the experiment. No significant changes were observed in TNF-α, IL-1β, and IL-6 m RNA levels as well as in the protein levels of CB2 receptors, glycolysis-associated enzymes PFK and LDHa. Additionally, immunofluorescent staining revealed no alteration in c-Fos protein expression (stained red) in cerebral cortex between AngII-induced mice and control mice.

1. The mRNA levels of TNFa, IL-1β and IL-6 had no significant change in cerebral cortex tissues between AngII-induced mice and control mice.

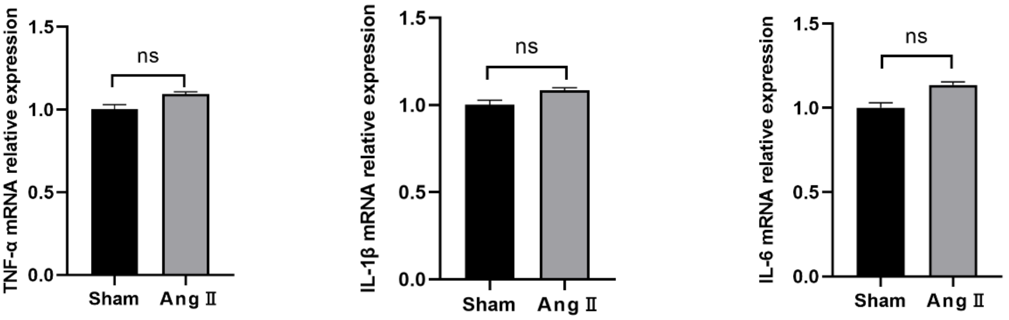

Figure S1. In the cerebral cortex tissues, there was no difference in the mRNA relative expression of TNFα, IL-1β, and IL-6 between the AngII-treated animals and the Sham mice. The results were expressed by mean±SEM (n=5 mice in each group, ns: $P > 0.05$).

2. There was no significant alteration in the protein levels of CB2 receptor in cerebral cortex tissues between AngII-induced mice and control mice.

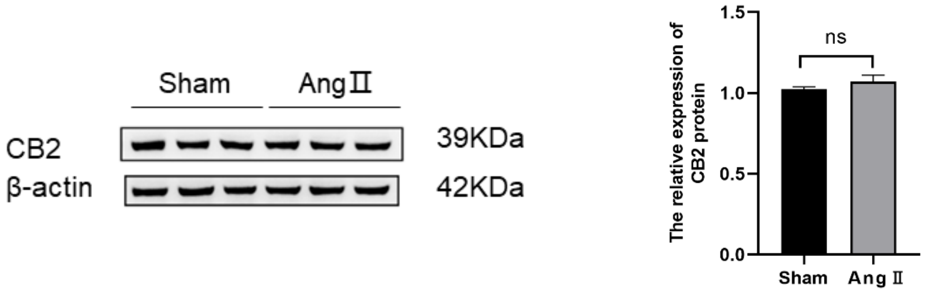

Figure S2. In the cerebral cortex tissues, there was no difference in the relative expression of CB2 receptor protein between AngII-treated and Sham-treated mice. The results were expressed by mean±SEM (n=5 mice in each group, ns: $P > 0.05$).

3. There was no significant alteration in the protein levels of glycolysis-associated enzymes PFK and LDHa in cerebral cortex tissues between AngII-induced mice and control mice.

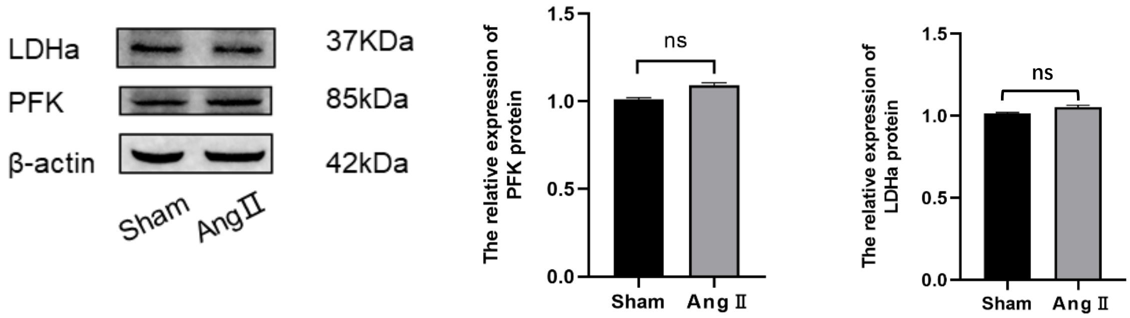

Figure S3. In the cerebral cortical tissues, there was no difference in the relative expression of PFK and LDHα proteins between AngII-treated and Sham-treated mice. The results were expressed by mean±SEM (n=5 mice in each group, ns: $P > 0.05$).

4. Immunofluorescent staining revealed no change in c-Fos protein expression (stained by red), indicating neuronal excitation, within the cerebral cortex of AngII-induced hypertensive mice; furthermore, no colocalization between c-Fos protein (stained by red) and Iba1-positive microglia (stained by blue) was observed.

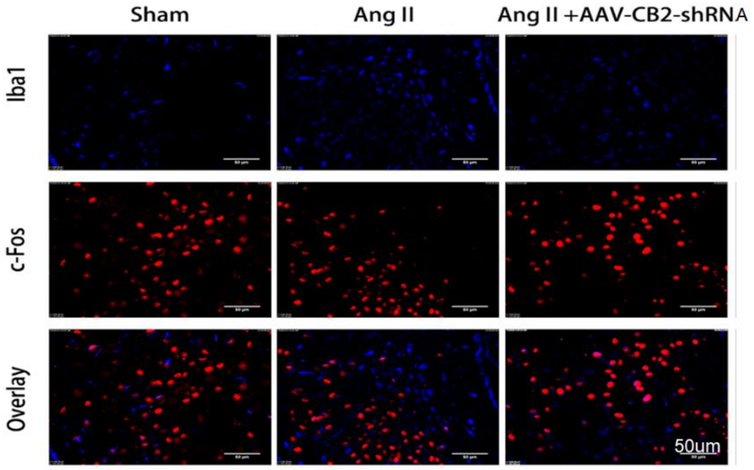

Figure S4. Immunofluorescent staining showed that there was no difference in the expression of the c-Fos protein (red, indicating neuronal excitation) between the Sham group, AngII+control-AAV, and AngII+ AAV-CB2-shRNA. Moreover, there was no colocalization between the red-stained c-Fos protein and the blue-stained Iba1-positive microglia among the three groups.

5. Blue dye was injected into the lateral ventricle.

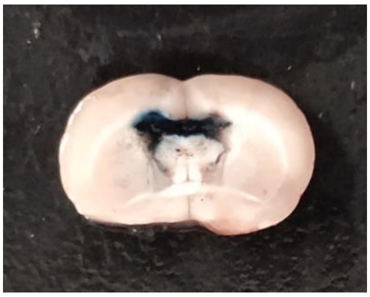

Figure S5. Blue dye was injected into the lateral ventricle.

6. Typical brain slice PVN region under a light microscope.

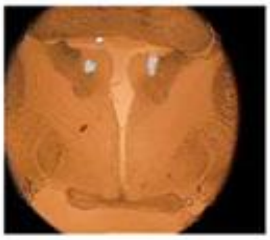

Figure S6. Typical brain slice PVN region under a light microscope.

7. The green fluorescent-labeled virus (stained by green) specifically targets microglial cells (Iba, stained by blue) in the brain.

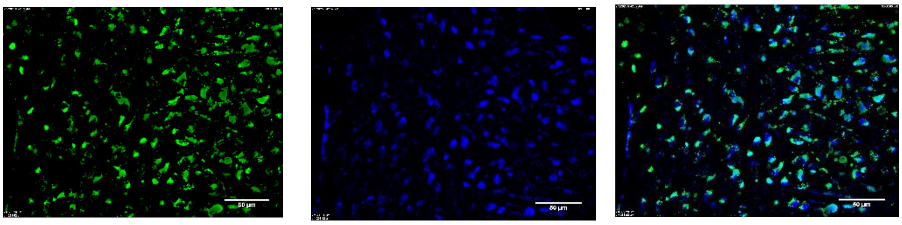

virus Iba Overlay

Figure S7. Immunofluorescence staining showed that the green fluorescent-labeled virus (green-stained) targets the brain's microglial cells (Iba, blue-stained).

8. In the BV2 cell experiments, 1μg/ml LPS has treated the BV2 for 24h, 36h and 48h. The mRNA expression of inflammation factors (TNFα, IL-1βand IL-6) were significantly increased in cells treated for 24h. So in the experiment, the cells treated by 1μg/ml LPS for 24h were selected.

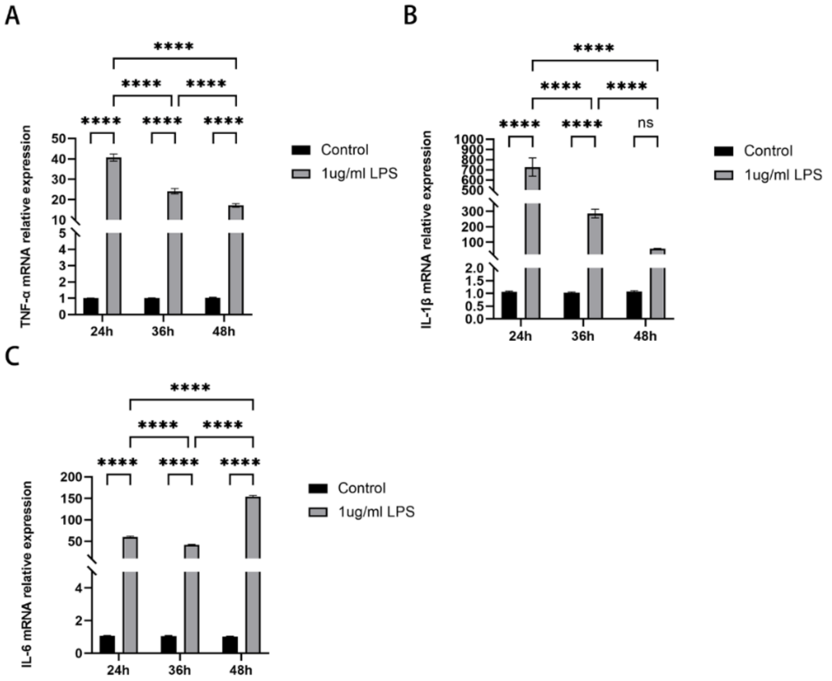

Figure S8. In the BV2 cell experiments, the effects of 1μg/ml LPS for 24h, 36h and 48h on the mRNA expression of inflammation factors TNFα(A), IL-1β (B) and IL-6 (C) were observed. The results were expressed by mean±SEM (n=5 mice in each group, ****P*<0.001, ns: *P*＞ 0.05)

9. In the BV2 cell experiments, the effects of 100nM and 1uM Ang Ⅱ for 24h, 36h and 48h on TNF-α mRNA, IL-1β mRNA and IL-6 mRNA in BV2 cells (RT-qPCR) were observed. The mRNA expression of inflammation factors (TNFα, IL-1βand IL-6) were significantly increased in 100nM Ang Ⅱ treated for 36h. So in the experiment, the cells treated by 100nM Ang Ⅱ for 36h were selected.

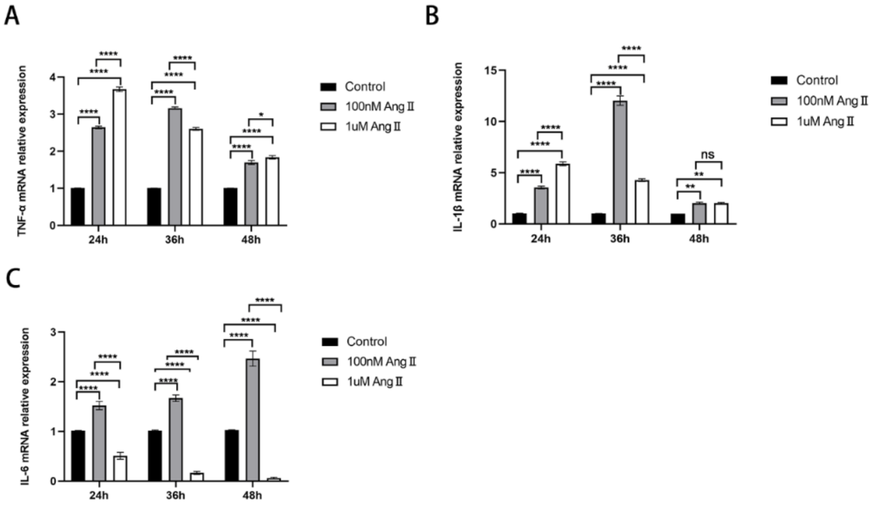

Figure S9. In the BV2 cell experiments, the effects of 100nM and 1uM Ang Ⅱ for 24h, 36h and 48h on TNF-α mRNA(A), IL-1β mRNA(B) and IL-6 mRNA (C) in BV2 cells were observed.

10. Table S1. Primers of genes were used in RT-qPCR

TNF-α	forward	ATGTCTCAGCCTCTTCTCATTC
TNF-α	reversed	GCTTGTCACTCGAATTTTGAGA
IL-1β	forward	CACTACAGGCTCCGAGATGAACAAC
IL-1β	reversed	TGTCGTTGCTTGGTTCTCCTTGTAC
IL-6	forward	CTCCCAACAGACCTGTCTATAC
IL-6	reversed	CCATTGCACAACTCTTTTCTCA
CB2	forward	TGATCCCTAACGACTACCTACT
CB2	reversed	TTTCCAGAGGACATACCCATAG
GAPDH	forward	GGTTGTCTCCTGCGACTTCA
GAPDH	reversed	TGGTCCAGGGTTTCTTACTCC
