# Supplementary figures and images for: Activation of Cannabinoid Type 2 Receptor in Microglia Reduces Neuroinflammation through Inhibiting Aerobic Glycolysis to Relieve Hypertension
Source: Biomolecules. 2024 Mar 11;14(3):333. doi: 10.3390/biom14030333 (PMC10967819; doi:10.3390/biom14030333)

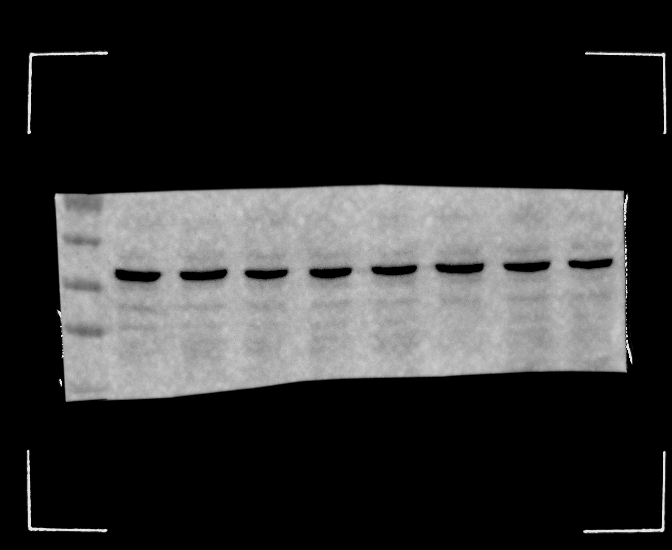

Supplement: Supplementary file 1 [file biomolecules-14-00333-s001.zip › biomolecules-2878643-Supplementary File S1 - original blot images/FIG1/ú¿aú⌐-1-b-actin.tif]

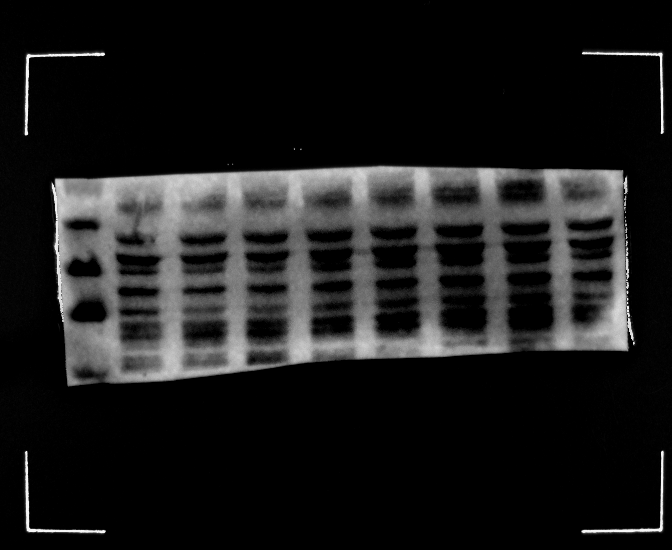

Supplement: Supplementary file 1 [file biomolecules-14-00333-s001.zip › biomolecules-2878643-Supplementary File S1 - original blot images/FIG1/ú¿aú⌐-1-CB2.tif]

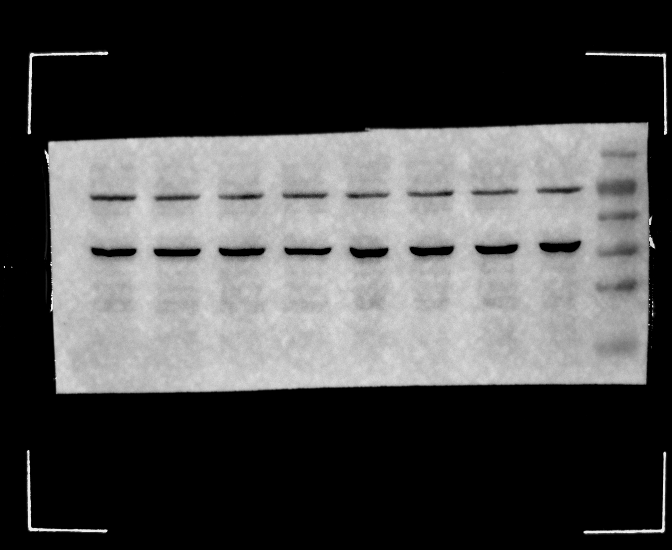

Supplement: Supplementary file 1 [file biomolecules-14-00333-s001.zip › biomolecules-2878643-Supplementary File S1 - original blot images/FIG1/ú¿aú⌐-2-b-actin.tif]

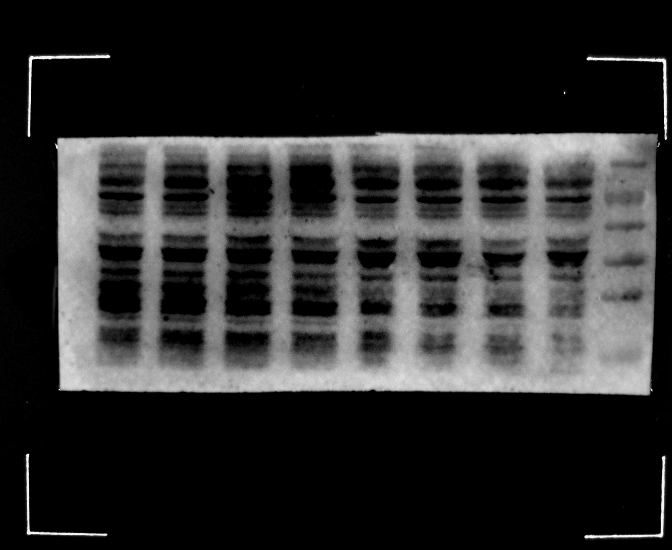

Supplement: Supplementary file 1 [file biomolecules-14-00333-s001.zip › biomolecules-2878643-Supplementary File S1 - original blot images/FIG1/ú¿aú⌐-2-CB2.tif]

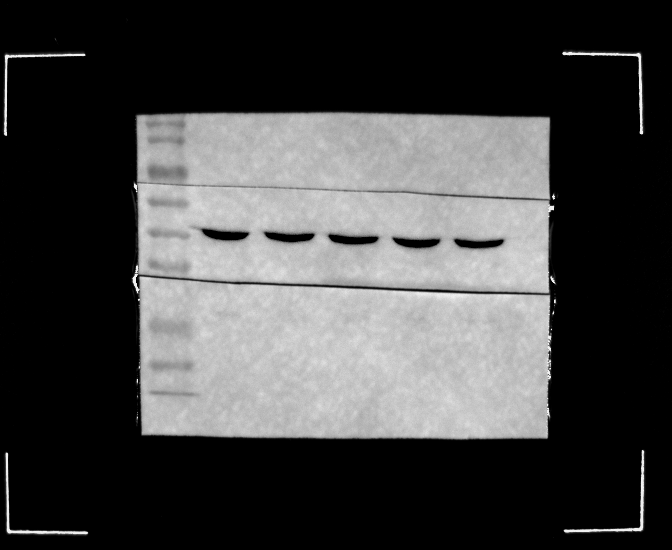

Supplement: Supplementary file 1 [file biomolecules-14-00333-s001.zip › biomolecules-2878643-Supplementary File S1 - original blot images/FIG1/ú¿dú⌐-1-b-actin.tif]

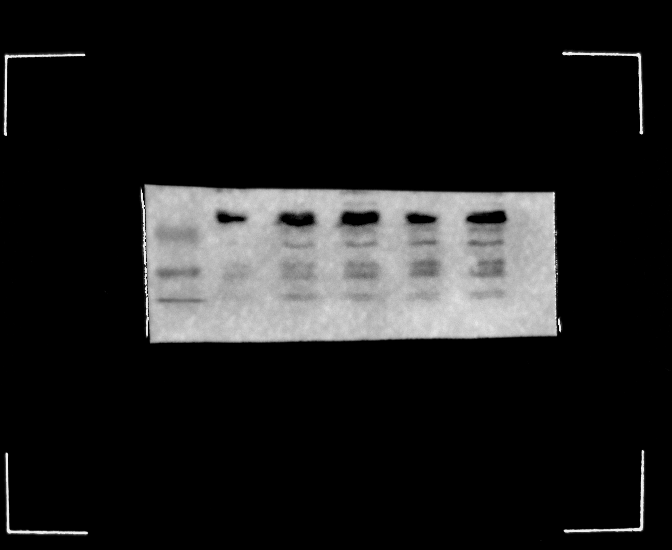

Supplement: Supplementary file 1 [file biomolecules-14-00333-s001.zip › biomolecules-2878643-Supplementary File S1 - original blot images/FIG1/ú¿dú⌐-1-TNF-a.tif]

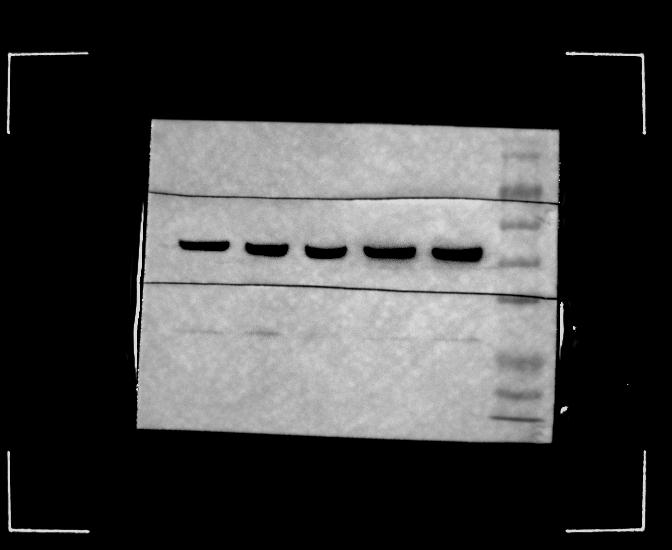

Supplement: Supplementary file 1 [file biomolecules-14-00333-s001.zip › biomolecules-2878643-Supplementary File S1 - original blot images/FIG1/ú¿dú⌐-2-b-actin.tif]

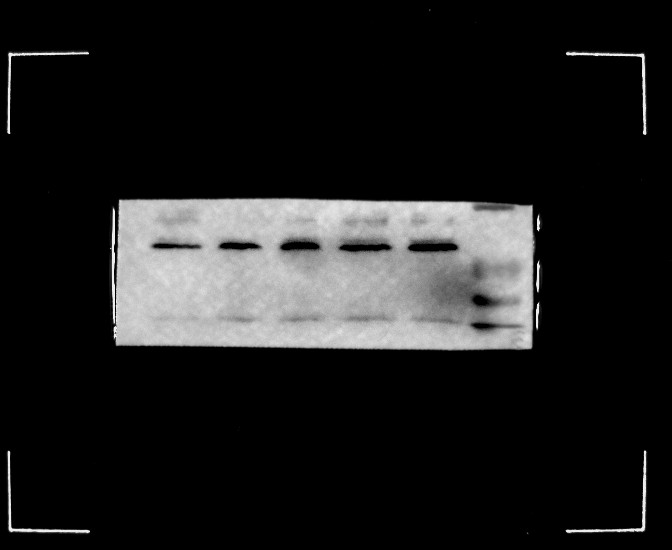

Supplement: Supplementary file 1 [file biomolecules-14-00333-s001.zip › biomolecules-2878643-Supplementary File S1 - original blot images/FIG1/ú¿dú⌐-2-il-1b.tif]

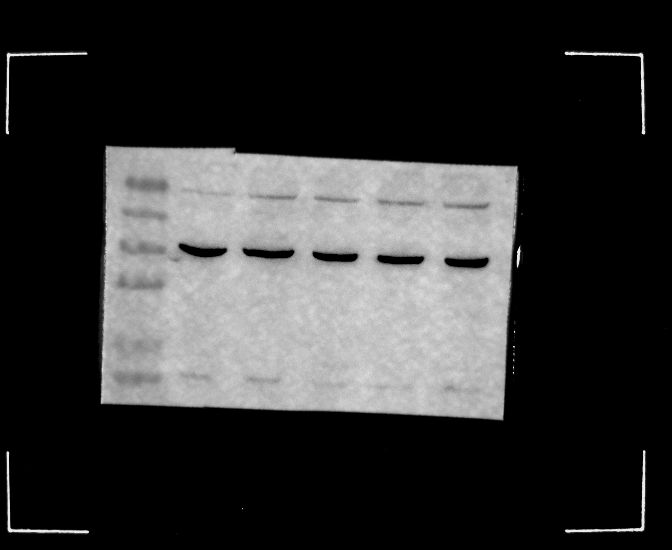

Supplement: Supplementary file 1 [file biomolecules-14-00333-s001.zip › biomolecules-2878643-Supplementary File S1 - original blot images/FIG1/ú¿dú⌐-3-b-actin.tif]

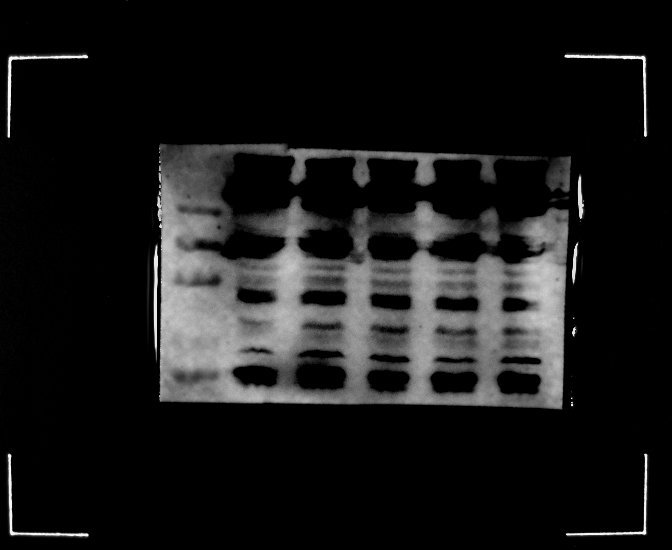

Supplement: Supplementary file 1 [file biomolecules-14-00333-s001.zip › biomolecules-2878643-Supplementary File S1 - original blot images/FIG1/ú¿dú⌐-3-IL-6.tif]

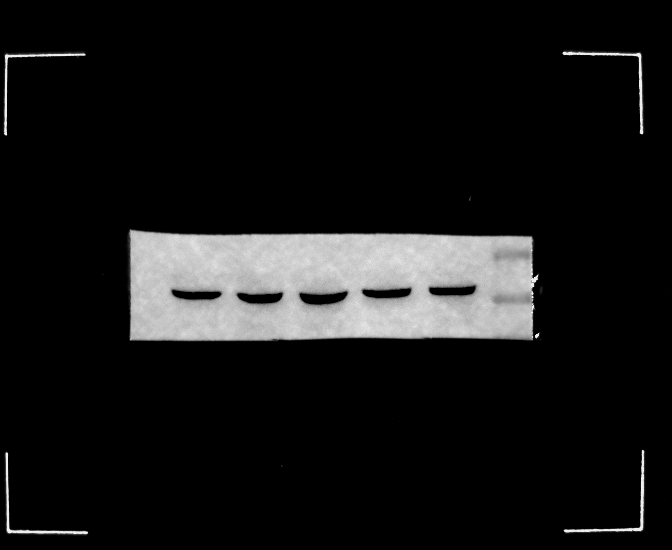

Supplement: Supplementary file 1 [file biomolecules-14-00333-s001.zip › biomolecules-2878643-Supplementary File S1 - original blot images/FIG1/ú¿eú⌐-1-b-actin.tif]

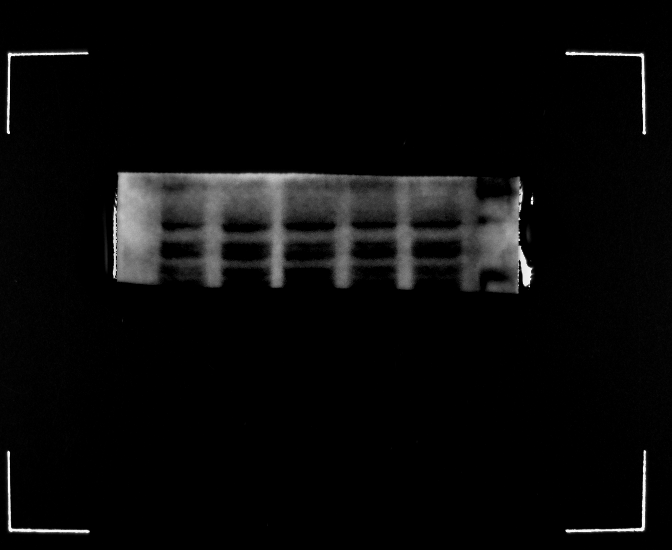

Supplement: Supplementary file 1 [file biomolecules-14-00333-s001.zip › biomolecules-2878643-Supplementary File S1 - original blot images/FIG1/ú¿eú⌐-1-PFK.tif]

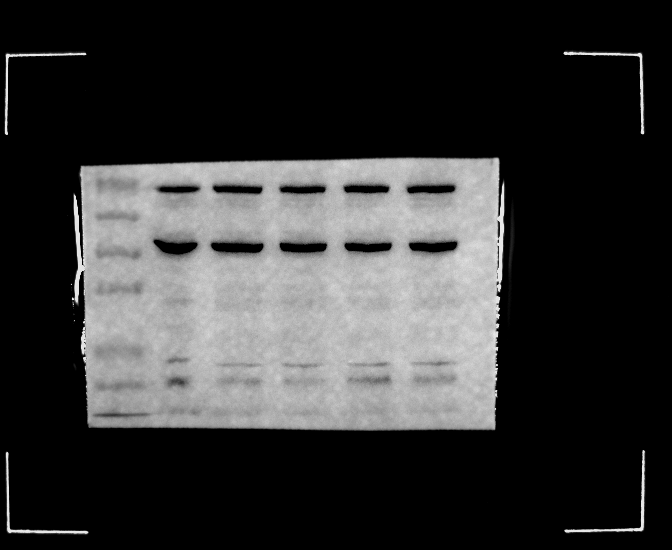

Supplement: Supplementary file 1 [file biomolecules-14-00333-s001.zip › biomolecules-2878643-Supplementary File S1 - original blot images/FIG1/ú¿eú⌐-2-b-actin.tif]

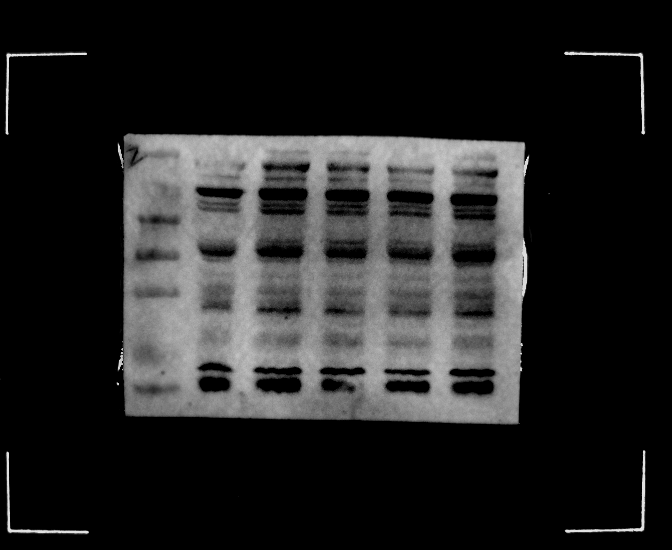

Supplement: Supplementary file 1 [file biomolecules-14-00333-s001.zip › biomolecules-2878643-Supplementary File S1 - original blot images/FIG1/ú¿eú⌐-2-LDHA.tif]

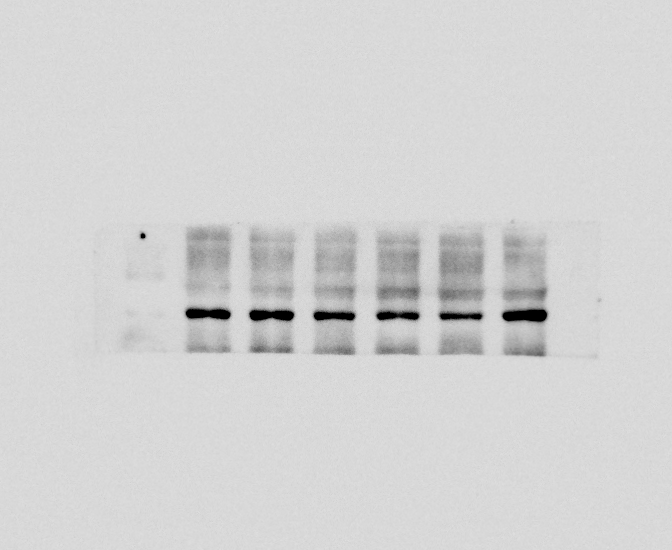

Supplement: Supplementary file 1 [file biomolecules-14-00333-s001.zip › biomolecules-2878643-Supplementary File S1 - original blot images/FIG2/ú¿dú⌐-1-b-actin.tif]

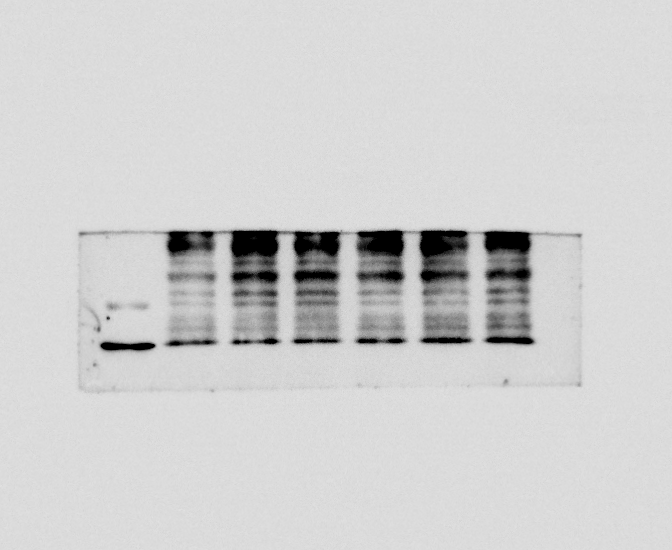

Supplement: Supplementary file 1 [file biomolecules-14-00333-s001.zip › biomolecules-2878643-Supplementary File S1 - original blot images/FIG2/ú¿dú⌐-1-IL-1B.tif]

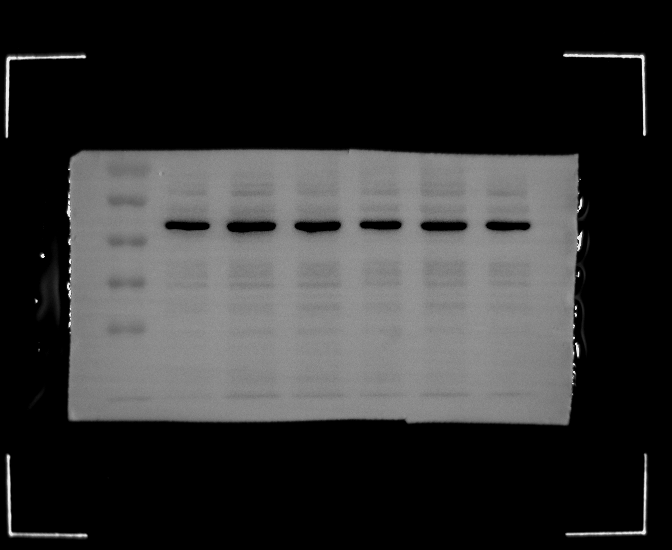

Supplement: Supplementary file 1 [file biomolecules-14-00333-s001.zip › biomolecules-2878643-Supplementary File S1 - original blot images/FIG2/ú¿dú⌐-2-b-actin .tif]

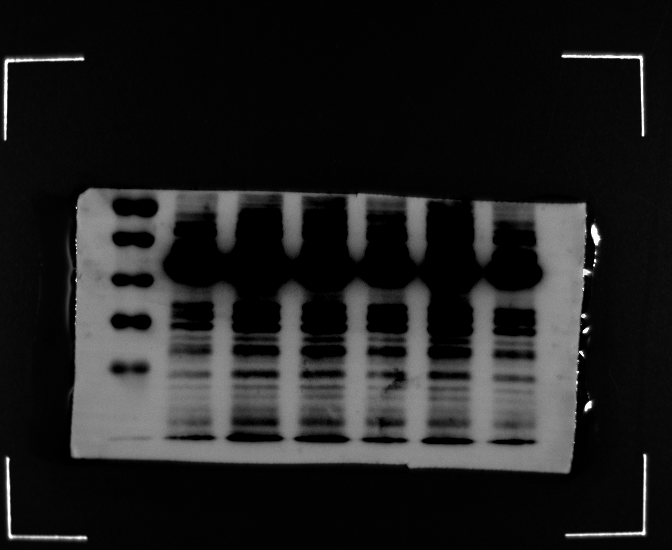

Supplement: Supplementary file 1 [file biomolecules-14-00333-s001.zip › biomolecules-2878643-Supplementary File S1 - original blot images/FIG2/ú¿dú⌐-2-il-6.tif]

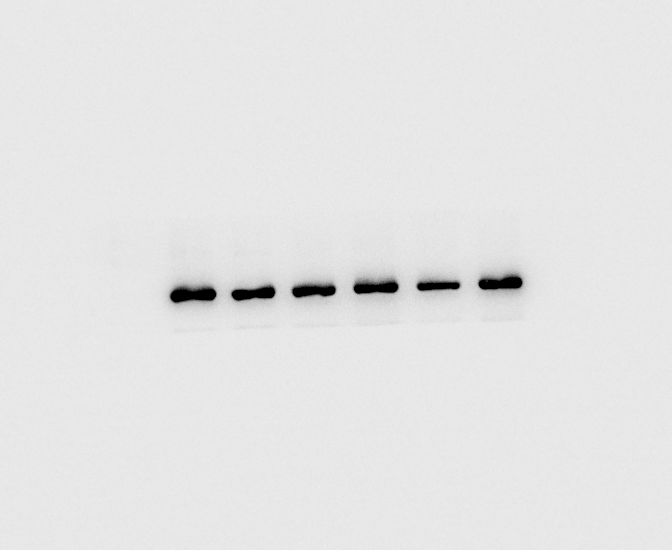

Supplement: Supplementary file 1 [file biomolecules-14-00333-s001.zip › biomolecules-2878643-Supplementary File S1 - original blot images/FIG2/ú¿dú⌐-3-b-actin.tif]

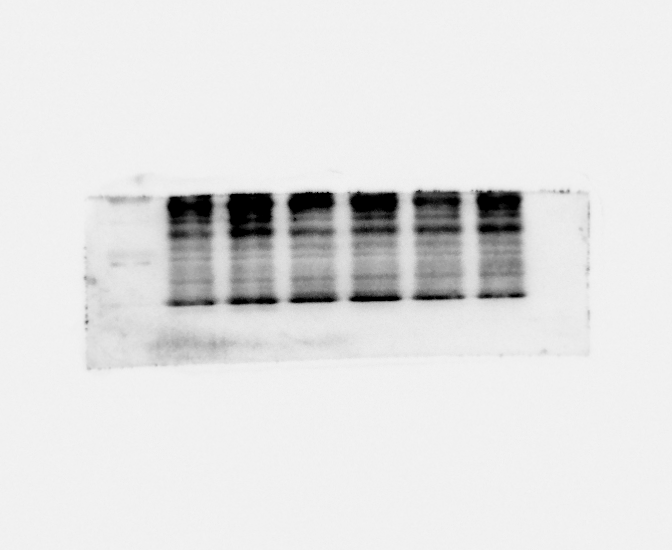

Supplement: Supplementary file 1 [file biomolecules-14-00333-s001.zip › biomolecules-2878643-Supplementary File S1 - original blot images/FIG2/ú¿dú⌐-3-TNF-a.tif]

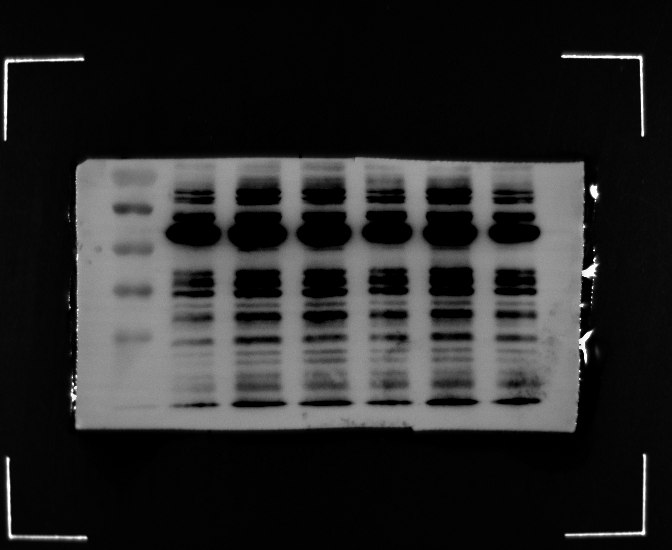

Supplement: Supplementary file 1 [file biomolecules-14-00333-s001.zip › biomolecules-2878643-Supplementary File S1 - original blot images/FIG3/(c)-1-TNF-a-.tif]

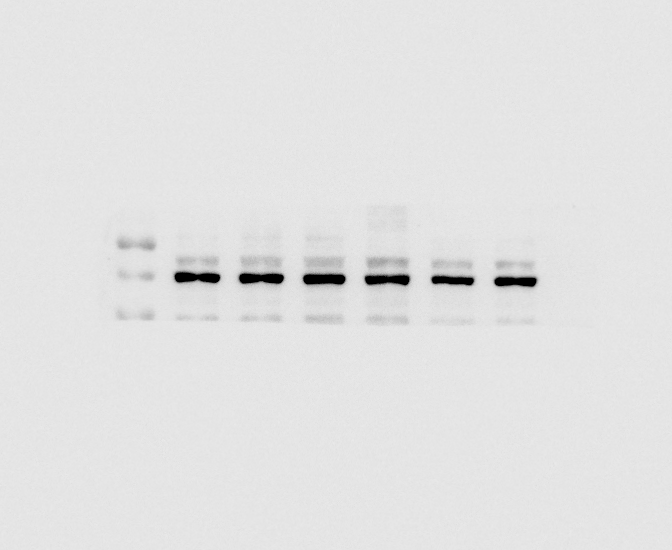

Supplement: Supplementary file 1 [file biomolecules-14-00333-s001.zip › biomolecules-2878643-Supplementary File S1 - original blot images/FIG3/(c)-2- b-actin.tif]

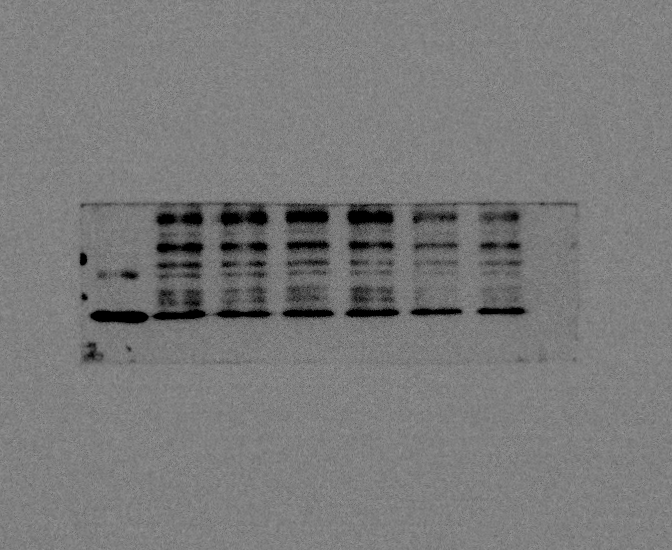

Supplement: Supplementary file 1 [file biomolecules-14-00333-s001.zip › biomolecules-2878643-Supplementary File S1 - original blot images/FIG3/(c)-2-IL-1b.tif]

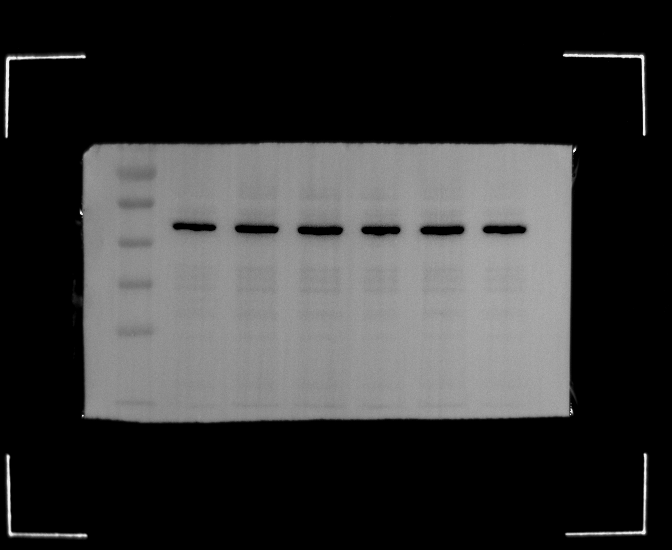

Supplement: Supplementary file 1 [file biomolecules-14-00333-s001.zip › biomolecules-2878643-Supplementary File S1 - original blot images/FIG3/(c)-3-b-actin.tif]

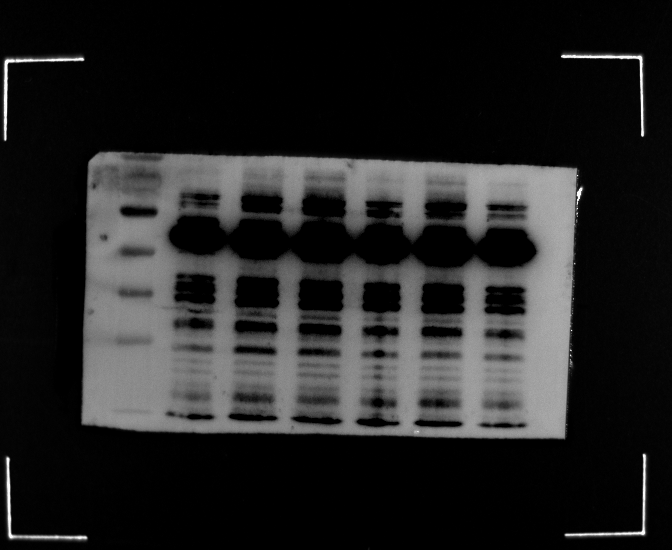

Supplement: Supplementary file 1 [file biomolecules-14-00333-s001.zip › biomolecules-2878643-Supplementary File S1 - original blot images/FIG3/(c)-3-il-6.tif]

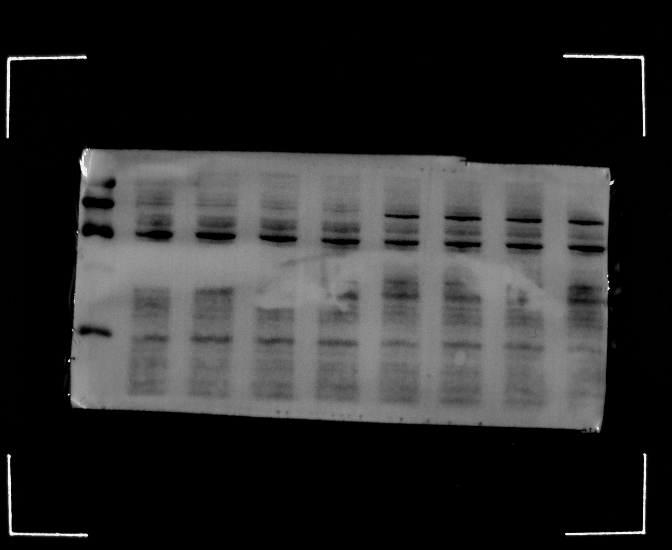

Supplement: Supplementary file 1 [file biomolecules-14-00333-s001.zip › biomolecules-2878643-Supplementary File S1 - original blot images/FIG3/ú¿aú⌐-b-actin.tif]

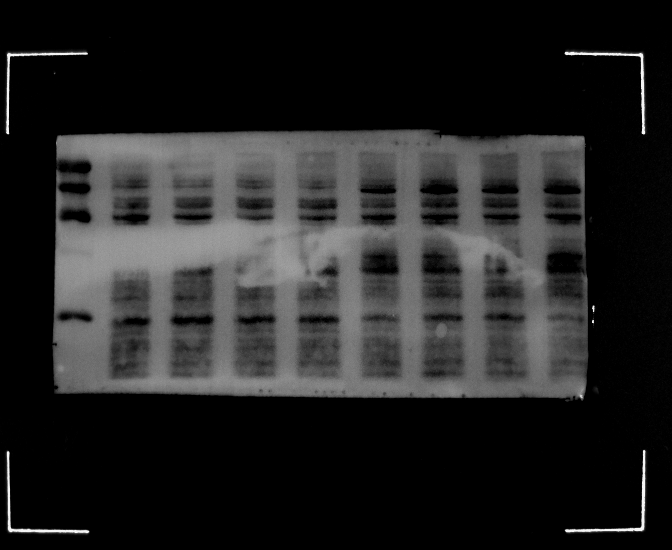

Supplement: Supplementary file 1 [file biomolecules-14-00333-s001.zip › biomolecules-2878643-Supplementary File S1 - original blot images/FIG3/ú¿aú⌐-CB2.tif]

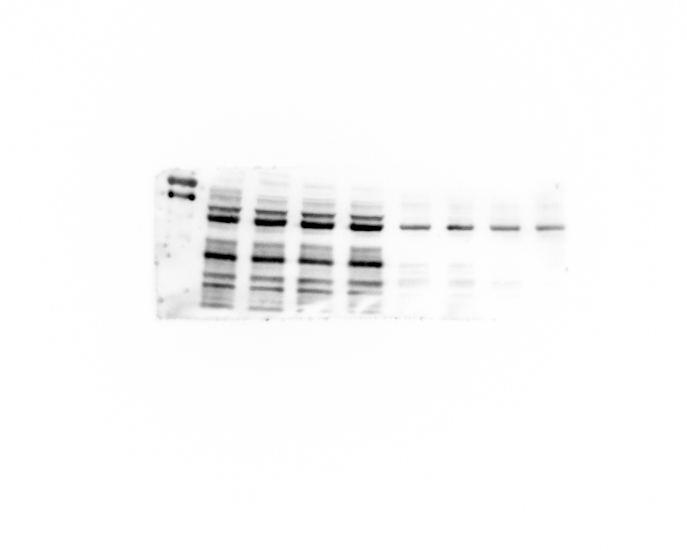

Supplement: Supplementary file 1 [file biomolecules-14-00333-s001.zip › biomolecules-2878643-Supplementary File S1 - original blot images/FIG3/ú¿b)-CB2.tif]

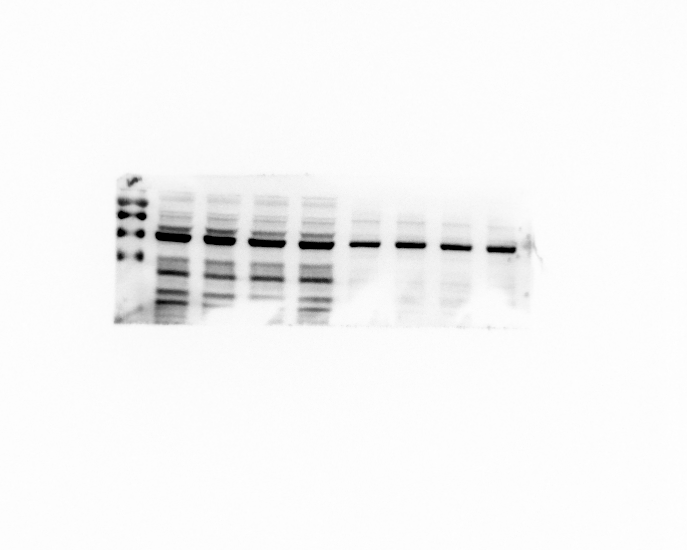

Supplement: Supplementary file 1 [file biomolecules-14-00333-s001.zip › biomolecules-2878643-Supplementary File S1 - original blot images/FIG3/ú¿bú⌐-b-actin.tif]

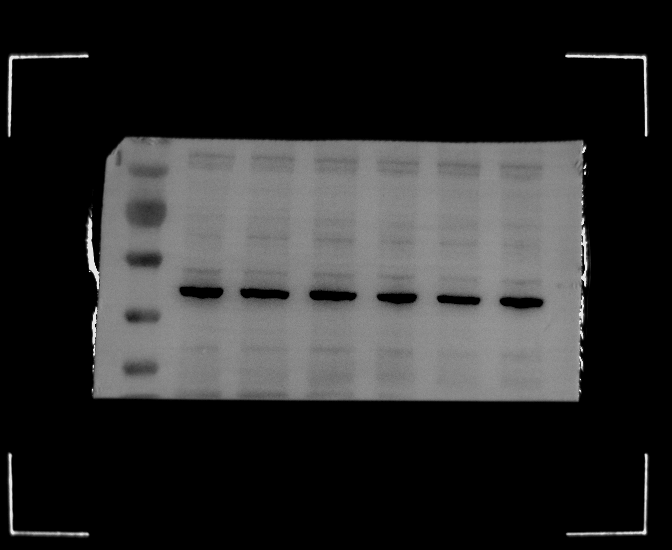

Supplement: Supplementary file 1 [file biomolecules-14-00333-s001.zip › biomolecules-2878643-Supplementary File S1 - original blot images/FIG4/(b)-1-b-actin.tif]

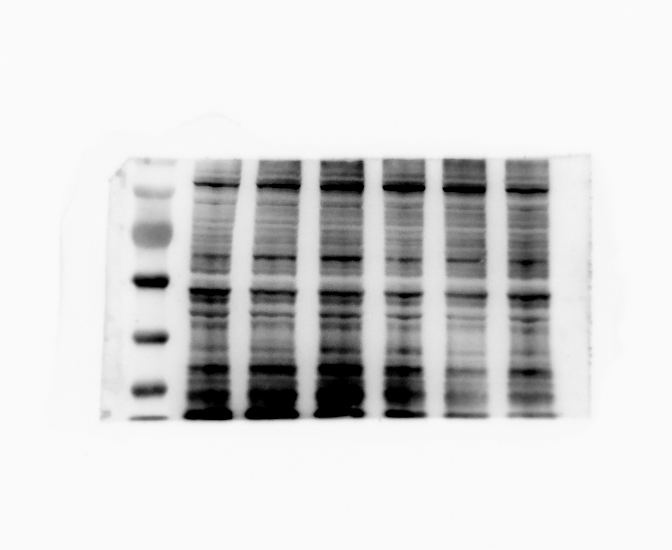

Supplement: Supplementary file 1 [file biomolecules-14-00333-s001.zip › biomolecules-2878643-Supplementary File S1 - original blot images/FIG4/(b)-1-PFK.tif]

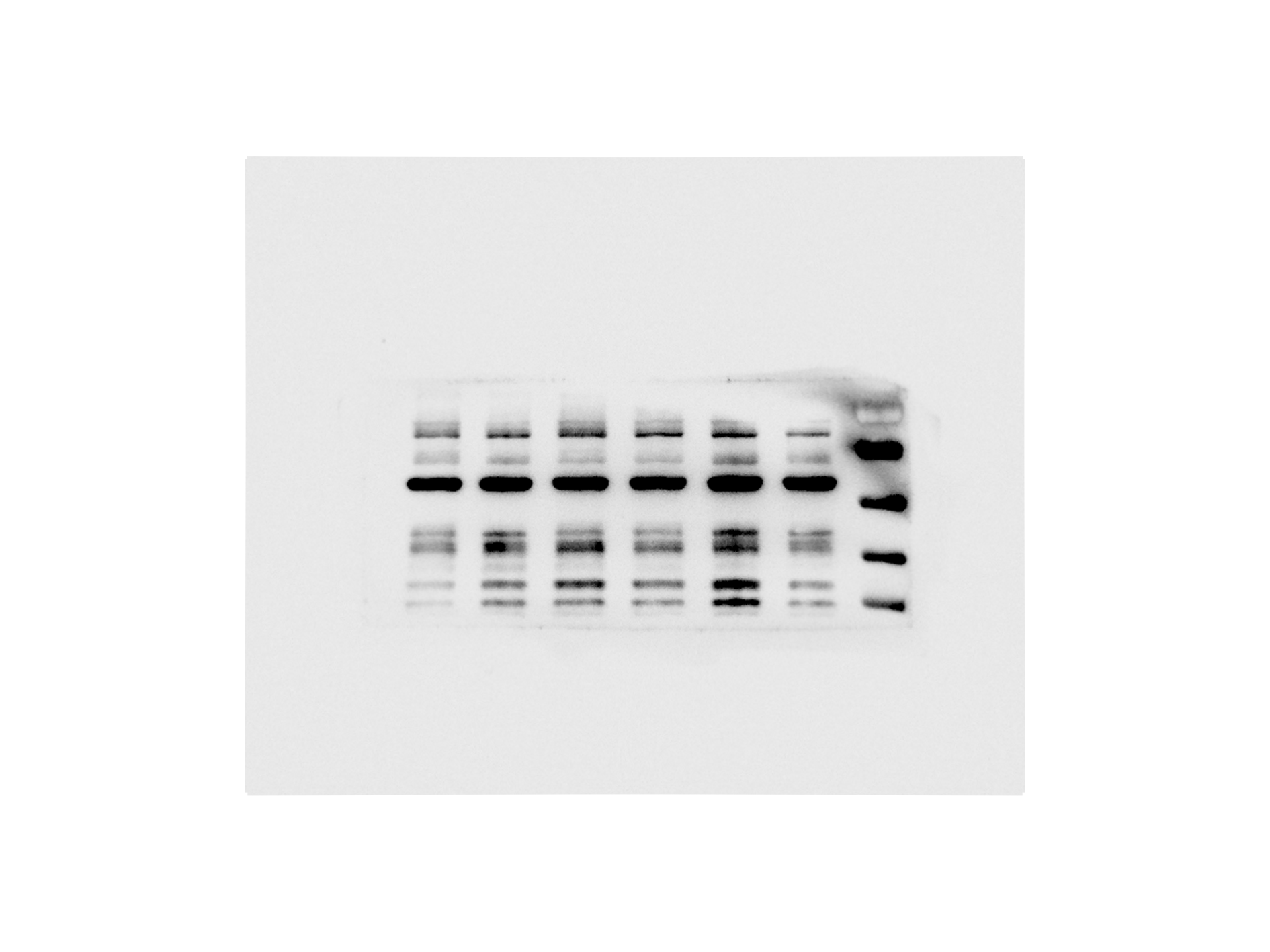

Supplement: Supplementary file 1 [file biomolecules-14-00333-s001.zip › biomolecules-2878643-Supplementary File S1 - original blot images/FIG4/(b)-2-b-actin.tif]

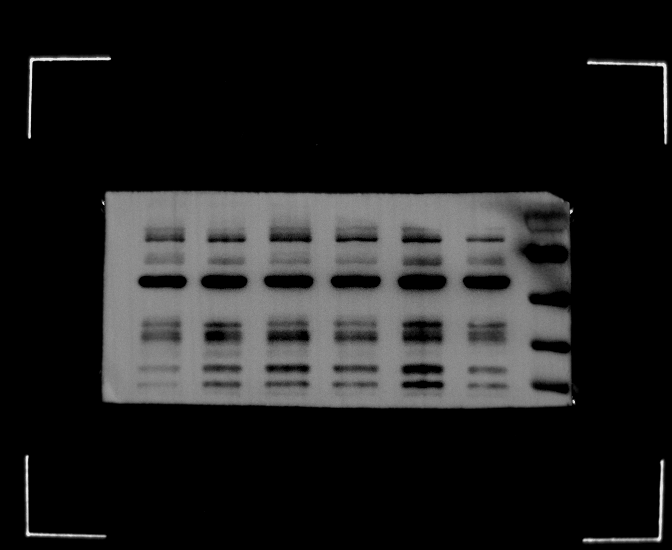

Supplement: Supplementary file 1 [file biomolecules-14-00333-s001.zip › biomolecules-2878643-Supplementary File S1 - original blot images/FIG4/(b)-2-LDHA.tif]

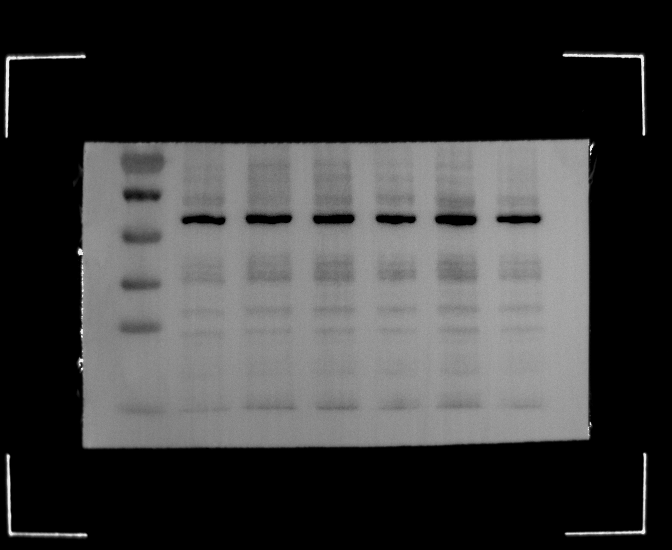

Supplement: Supplementary file 1 [file biomolecules-14-00333-s001.zip › biomolecules-2878643-Supplementary File S1 - original blot images/FIG5/(b)-b-actin.tif]

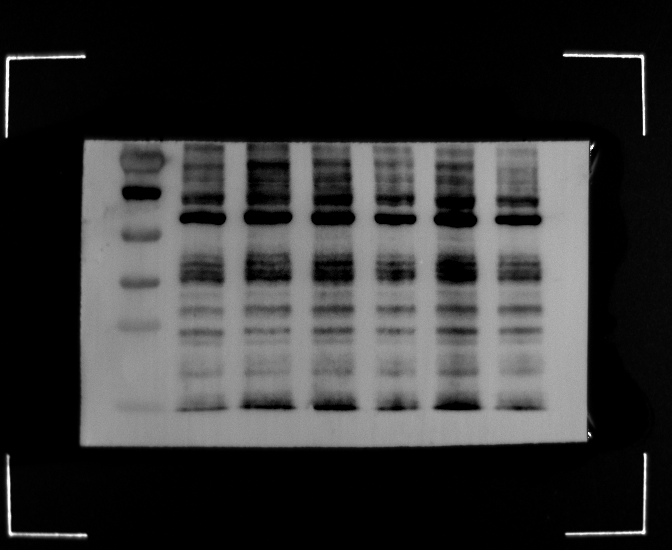

Supplement: Supplementary file 1 [file biomolecules-14-00333-s001.zip › biomolecules-2878643-Supplementary File S1 - original blot images/FIG5/(b)-TNF-a.tif]

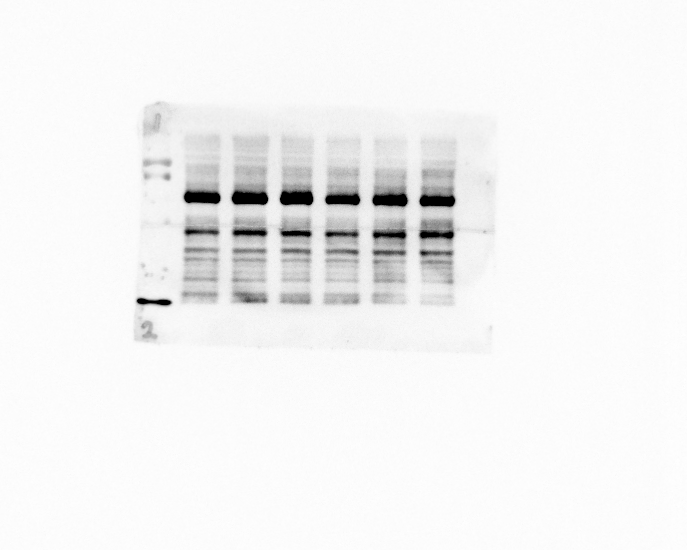

Supplement: Supplementary file 1 [file biomolecules-14-00333-s001.zip › biomolecules-2878643-Supplementary File S1 - original blot images/FIG5/(c)b-actin+il-b.tif]

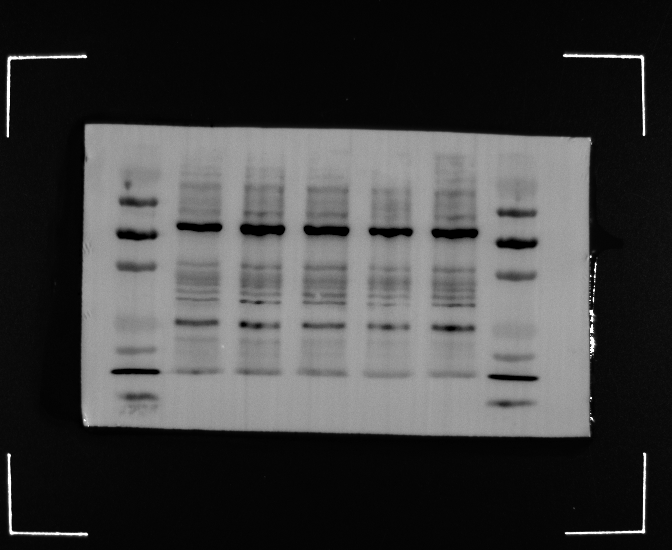

Supplement: Supplementary file 1 [file biomolecules-14-00333-s001.zip › biomolecules-2878643-Supplementary File S1 - original blot images/FIG5/ú¿dú⌐-b-actin.tif]

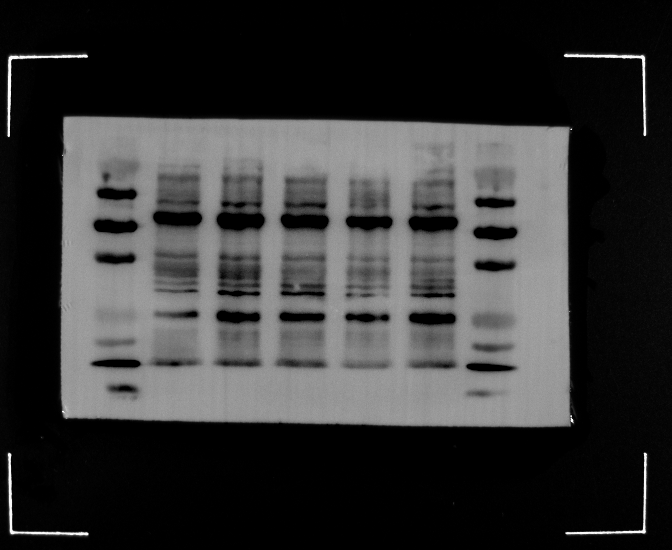

Supplement: Supplementary file 1 [file biomolecules-14-00333-s001.zip › biomolecules-2878643-Supplementary File S1 - original blot images/FIG5/ú¿dú⌐-IL-6.tif]
